# The WRKY transcription factor HpWRKY44 regulates *CytP450-like1* expression in red pitaya fruit (*Hylocereus polyrhizus*)

**DOI:** 10.1038/hortres.2017.39

**Published:** 2017-08-02

**Authors:** Mei-nv Cheng, Zi-juan Huang, Qing-zhu Hua, Wei Shan, Jian-fei Kuang, Wang-jin Lu, Yong-hua Qin, Jian-ye Chen

**Affiliations:** 1 State Key Laboratory for Conservation and Utilization of Subtropical Agro-bioresources/Guangdong Provincial Key Laboratory of Postharvest Science of Fruits and Vegetables/Key Laboratory of Biology and Genetic Improvement of Horticultural Crops-South China, Ministry of Agriculture, College of Horticulture, South China Agricultural University, Guangzhou 510642, China

## Abstract

Red pitaya (*Hylocereus polyrhizus*) fruit is a high-value, functional food, containing a high level of betalains. Several genes potentially related to betalain biosynthesis, such as cytochrome P450-like (*CytP450-like*), have been identified in pitaya fruit, while their transcriptional regulation remains unclear. In this work, the potential involvement of a WRKY transcription factor, HpWRKY44, in regulating *CytP450-like1* expression in pitaya fruit was examined. HpWRKY44, a member of the Group 1 WRKY family, contains two conserved WRKY motifs and is localized in the nucleus. HpWRKY44 also exhibits trans-activation ability. Gene expression analysis showed that the expression of *HpCytP450-like1* and *HpWRKY44* increased steadily during pitaya fruit coloration, which corresponded with the production of elevated betalain levels in the fruit. HpWRKY44 was also demonstrated to directly bind to and activate the *HpCytP450-like1* promoter via the recognition of the W-box element present in the promoter. Collectively, our findings indicate that HpWRKY44 transcriptionally activates *HpCytP450-like1*, which perhaps, at least in part, contributes to betalain biosynthesis in pitaya fruit. The information provided in the current study provides novel insights into the regulatory network associated with betalain biosynthesis during pitaya fruit coloration.

## Introduction

Betalains are naturally occurring, water-soluble pigments produced from amino acid L-tyrosine. More than 50 natural betalains have been reported and they are present only in species within the order Caryophyllales, such as beets, *Amaranthus*, prickly pear cactus and red pitaya fruit.^1–3^ The red-violet betacyanins and the yellow betaxanthins, are the two types of betalains.^4^ These pigments are not produced in the same plant or at the same time when anthocyanins, another major pigment group, are present.^5^ Betalains are widely used as economically important natural food colorants and functional foods.^6,7^ Therefore, understanding the regulatory mechanisms responsible for betalain biosynthesis could provide information to assist the development of biotechnological approaches aimed to improve betalain production in plants.

The biochemical pathway of betalain biosynthesis from tyrosine has been extensively studied.^1,8–10^ Enzymes involved in betalain biosynthesis, such as tyrosinase (TYR), 4,5-dihydroxy-phenylalanine (DOPA)-dioxygenase (DOD) and glucosyltransferases (GTs) have been identified in higher plants.^1,8–10^ It has been proposed that TYR converts tyrosine to L-3,4-dihydroxyphenylalanine (L-DOPA), which in turn is converted to betalamic acid by DOD. GTs, the last family of enzymes in the pathway, make betalains stable and diversify their composition by transferring sugar residues.^1,8–10^ Three cytochrome P450-type enzymes, CYP76AD1, CYP76AD5 and CYP76AD6, have been well documented to be associated with the hydroxylation of tyrosine to form L-DOPA. In addition, CYP76AD1 has also been shown to function exclusively in the formation of 5,6-dihydroxyindoline-2-carboxylic acid (*cyclo*-DOPA) from L-DOPA in red beet.^9,11–13^ Genes encoding these enzymes have been identified in plant species within the Caryophyllales and their expression levels have been demonstrated to increase with betalain accumulation, suggesting that betalain biosynthesis is regulated by the transcription level of the genes encoding these enzymes.^1^ Recently, a betalain biosynthetic gene, *CYP76AD1* has been demonstrated to be transcriptional regulated by a MYB-family transcription factor (TF).^14^ This is the first TF reported to be involved in the regulation of the betalain biosynthesis. Although thousands of TFs have been identified in plants, however, the identification and role of other TFs regulating betalain biosynthesis remains largely unknown.

As a large family of plant-specific TFs, the first WRKY protein is identified in sweet potato.^15^ Numerous *WRKY* genes have now been isolated and characterized in many plants. For instance, >197, 100, 74, 287 and 62 WRKY members have been identified in *Glycine max, Oryza sativa*, *Arabidopsis*, *Brassica napus* and *Fragaria vesca,* respectively.^16–20^ Two highly conserved domains are found in WRKY proteins, one is WRKY with WRKYGQK sequences, and the other is a C-terminal zinc-finger motif of Cys and His residues.^16,21^ Plant WRKY proteins are well-known to be involved in various stress responses, hormone signaling, morphogenesis of trichomes and embryos, and senescence.^20,22–24^ WRKYs are also reported to regulate plant metabolite biosynthesis, including phenylpropanoids, alkaloids and terpenes, by regulating metabolite biosynthetic genes.^25–28^ Betalains are also secondary metabolites, however, the involvement of WRKY TFs in betalain biosynthesis, especially in economical fruits, has been largely unexplored.

Pitayas, also called as dragon fruit, belongs to the genus *Hylocereus* in the order Caryophyllales, and is commercially cultivated due to its nutritional value.^29^ There are several species of pitayas, while the most widely cultivated are *Hylocereus undatus* and *Hylocereus polyrhizus*, which have white and red colored pulp, respectively.^30^ As red pulp pitaya contains high levels of betalains, most of the previous studies of this fruit have focused on purification and characterization of betalins.^30,31^ Recently, several putative genes, including *TYR*, *DOD-like*, *CytP450-like* and *GT-like,* were identified in red pulp pitaya using RNA-seq, and associated with betalain biosynthesis.^10^ In the present work, the possible association of a Group I WRKY TF, HpWRKY44, with the direct activation of *HpCytP450-like1* is reported, providing new information on the transcriptional control of betalain production in pitaya fruit.

## Materials and methods

### Fruit samples

Red pitaya plants (*Hylocereus polyrhizus* cv. Dayeshuijing) were grown under field conditions at a local commercial plantation in Guangzhou, China. Fruits were sampled on the 16^th^, 21^st^, 26^th^, 30^th^, 35^th^, 40^th^ and 49^th^ day after artificial pollination (DAAP). The sample dates were selected to provide a set of samples covering pulp color changes in the fruit. At each sampling, internal fruit tissue (pulp tissue) from three fruits from three different plants were sampled, sliced, and frozen in liquid nitrogen immediately. Samples were stored at −80 °C for future analysis.

### Betalain quantification

The level of betalains in the pulp tissue of each sample was determined as previously described.^10^ Betalains were isolated from 0.5 g pulp with 5 mL 80% aqueous methanol (v/v) solution. The amount of betacyanin and betaxanthin in the solutions were measured by spectrophotometry (Infinite M200, Tecan Co.) at 538 and 483 nm, respectively, and reported as mg/100 g fresh pulp.

### Gene isolation and sequence analysis

Total RNA was extracted from pitaya pulp samples using the Quick RNA Isolation Kit (Huayueyang, Beijing, China) following manufacturer’s instructions. RNA quality was assessed by gel electrophoresis and spectrophotometry. The extracted RNA was used as template to synthesis cDNAs using a PrimeScript RT reagent Kit with gDNA Eraser (TaKaRa, shiga, Japan).

On the basis of the generation of a previous RNA-seq database,^10^ a WRKY TF was found to be significantly up-regulated during pulp coloration. As this WRKY displays high degree of sequence homology to *Arabidopsis thaliana* WRKY44, thus it was termed as *HpWRKY44*. *HpWRKY44* was cloned (primers are listed in Supplementary Table S1), sequenced and the resulting sequence was queried against the NCBI database in order to identify homologous genes. The theoretical isoelectric point (pI) and mass value for HpWRKY44 protein were determined following the method described at http://web.expasy.org/compute_pi/. Sequences were aligned using CLUSTALW (version 1.83) and GeneDoc software. A phylogenetic tree was constructed using the Neighbor–Joining method in MEGA5.0.

### Gene expression analysis

Gene expression was analyzed by reverse transcription-quantitative PCR (RT-qPCR) using GoTaq qPCR Master Mix Kit (Promega, Madison, WI, USA) as described previously.^10^ The PCR was conducted on a Bio-Rad CFX96 Real-Time PCR System. The cycling began with an initial denaturation step at 94 °C for 5 min, followed by 40 cycles of 94 °C for 10 s, 60 °C for 30 s, and 72 °C for 30 s. A no-template control and melting curve analysis was included in every PCR run. Relative gene expression was normalized according to the cycle threshold (*C*t) value using *actin* as the reference gene. Primers used in the RT-qPCR analysis are listed in Supplementary Table S1.

### Subcellular localization of HpWRKY44

To determine the cellular localization of *HpWRKY44*, its full-length was cloned into pEAQ-GFP for fusion with a *GFP* reporter gene (primers are listed in Supplementary Table S1). The *Agrobacterium tumefaciens* strain GV3101 cells carrying pEAQ-HpWRKY44-GFP or the GFP positive control were infiltrated into tobacco (*Nicotiana benthamiana*) leaves as previously described.^32,33^ Transient expression of GFP was recorded using a fluorescence microscope (Zeiss Axioskop 2 Plus) at 500 nm after two days of infiltration.

### *HpCytP450-like1* promoter analysis

A CTAB-based method was used to extract genomic DNA from pitaya pulps.^34^ The *HpCytP450-like1* promoter was isolated with a Genome Walker Kit (Clontech) using a nested PCR approach. The sequence of *HpCytP450-like1* promoter was subjected to the Plant-CARE database (http://bioinformatics.psb.ugent.be/webtools/plantcare/html/) for the prediction of conserved *cis*-element motifs. Specific primers are listed in Supplementary Table S1.

### Electrophoretic mobility shift assay

The N-terminus of *HpWRKY44,* including the WRKY domain (from 574 to 1194 bp), was inserted into pGEX-4T-1 (GE Healthcare Life Sciences (China), Beijing, China) to construct a GST-HpWRKY44 expression vector, and introduced into *Escherichia coli* strain BM Rosetta (DE3). GST-HpWRKY44 fusion protein was purified using Glutathione-Superflow Resin (Clontech, California, United States) after induction by 1 mM isopropyl thio-β-D-galactoside (IPTG) at 30 °C for 6 h.

LightShift Chemiluminescent EMSA Kit (Thermo Scientific, Illinois, United States) was used to perform EMSA as previously described.^33,35^ DNA fragments, including an oligonucleotide harboring two W-box (TTGAC) motifs and the *HpCytP450-like1* promoter with 74 bp containing two consensus W-box motifs were biotin-labeled at the 5′ end (primers are listed in Supplementary Table S1). The same or mutated probes without biotin labeling were used as the competitors. These probes were incubated with purified GST-HpWRKY44 fusion protein, and the GST protein alone was used as the negative control. After SDS–PAGE separation, protein-DNA complexes were detected using the chemiluminescence method and photos were taken on a ChemiDoc MP Imaging System (Bio-Rad, Hercules, CA, USA).

### Transcriptional activity

Dual-luciferase transient expression system in tobacco leaves was adopted to investigate the transcriptional activity of HpWRKY44. The reporter vector was constructed based on the pGreenII 0800-LUC vector.^36^ The minimal TATA box of the CaMV 35S promoter plus five copies of the GAL4 DNA-binding element (5×GAL4) were placed in front of the firefly luciferase (LUC). CaMV 35S-driving Renilla luciferase (REN) in the same vector was considered as an internal control. For the effector vector, the full-length of *HpWRKY44* fusing with the yeast GAL4 DNA-binding domain (GAL4BD) was droven by CaMV 35S.

In order to assess the specific binding and activity of HpWRKY44 to *HpCytP450-like1* promoter, the *HpCytP450-like1* promoter (1532 bp) was inserted into the pGreenII0800-LUC vector,^36^ while HpWRKY44 was inserted into the pEAQ vector as an effector. Primers for all constructs are listed in Supplementary Table S1.

A total of 2.5 μg plasmid DNA of the reporter and the effector were co-transformed into tobacco leaves in each assay, as described above. Two days after inoculation, LUC and REN luciferase activity was quantified as described previously.^33^ The transcriptional activity of HpWRKY44, as well as the trans-activation of *HpCytP450-like1* by the HpWRKY44, were calculated as the ratio of LUC/REN. For each pair, at least six independent replicates were assessed.

### Data analysis

At least three individual biological replicates were utilized in all of the conducted analyses. Data represent the mean±s.e.m. A one-way ANOVA was performed to determine the significance of experimental means at *P*<0.05 and *P*<0.01.

## Results and discussion

### Changes in betacyanin and betaxanthin content during color development of pitaya fruit pulp color

**Figure 1:**
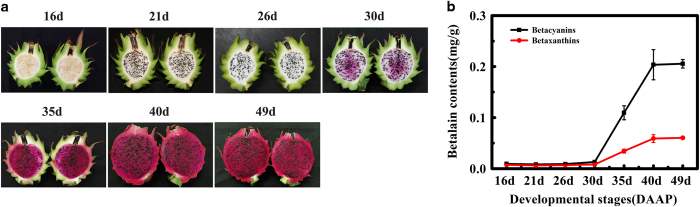
Pitaya fruit pulp at different developmental stages (**a**) and changes in betalain content (**b**) during fruit pulp color formation. Fruits were sampled for subsequent analyses at 16^th^, 21^st^, 26^th^, 30^th^, 35^th^, 40^th^ and 49^th^ days after artificial pollination (DAAP). Data represent mean±s.e.m. of three biological replicates (*n*=3).

In pitaya fruit there are two types of betalains which are reddish violet (betacyanins) and yellow (betaxanthins).^1^ As illustrated in Figures 1a and b, both betacyanin and betaxanthin content began to increase with the onset of color development in the pulp beginning at 30 DAAP. Levels steadily increased afterwards and the whole pulp was deep red by 49 DAAP.

### Promoter analysis of *HpCytP450-like1*

Previous transcriptomic and RT-qPCR analysis revealed that among the identified putative genes that are potentially related to betalain biosynthesis, the expression level of two *CytP450* genes (*HpCytP450-like1* and *HpCytP450-like4*), and one *DOD* gene (*DODA-like2*), increased gradually during the development of the red pulp color, and that the gradual increase in the expression of these genes corresponded with the accumulation of betalain during the transition of pulp color from white to red.^10^ A database query indicated that the amino acid sequence of HpCytP450-like1 and DODA-like2 shared high identity with BvCYP76AD1 (74%) and BvDODA1 (63%), respectively; two genes that are known to be involved in betalains biosynthesis in red sugar beet.^9,14^ These homologies indicated that *HpCytP450-like1* and *DODA-like2* might be related to betalains biosynthesis in pitaya fruit. Further biochemical and molecular evidence are required, however, to support their direct involvement in betalain biosynthesis, and to identify where they belong in the betalain biosynthetic pathway. In the current study, an attempt was made to isolate the promoters of all three of these genes, however, only the cloning of the *HpCytP450-like1* promoter was successful. A motif scan of the 1532 bp-long *HpCytP450-like1* promoter was conducted using Plant-CARE database in order to identify *cis*-acting elements. In addition to the core *cis*-acting elements, such as a TATA box and CAAT box, three typical W-box motifs with a core sequence (C/T)TGAC(C/T) (Supplementary Text S1) were identified in the *HpCytP450-like1* promoter. The W-box is a well-known binding element of WRKY TFs, suggesting the possible involvement of WRKY TFs in regulating *HpCytP450-like1*. Binding sites for MYB, HSF and AREB/ABF TFs were also identified (Table 1).

### Cloning and sequence analysis of HpWRKY44

**Figure 2:**
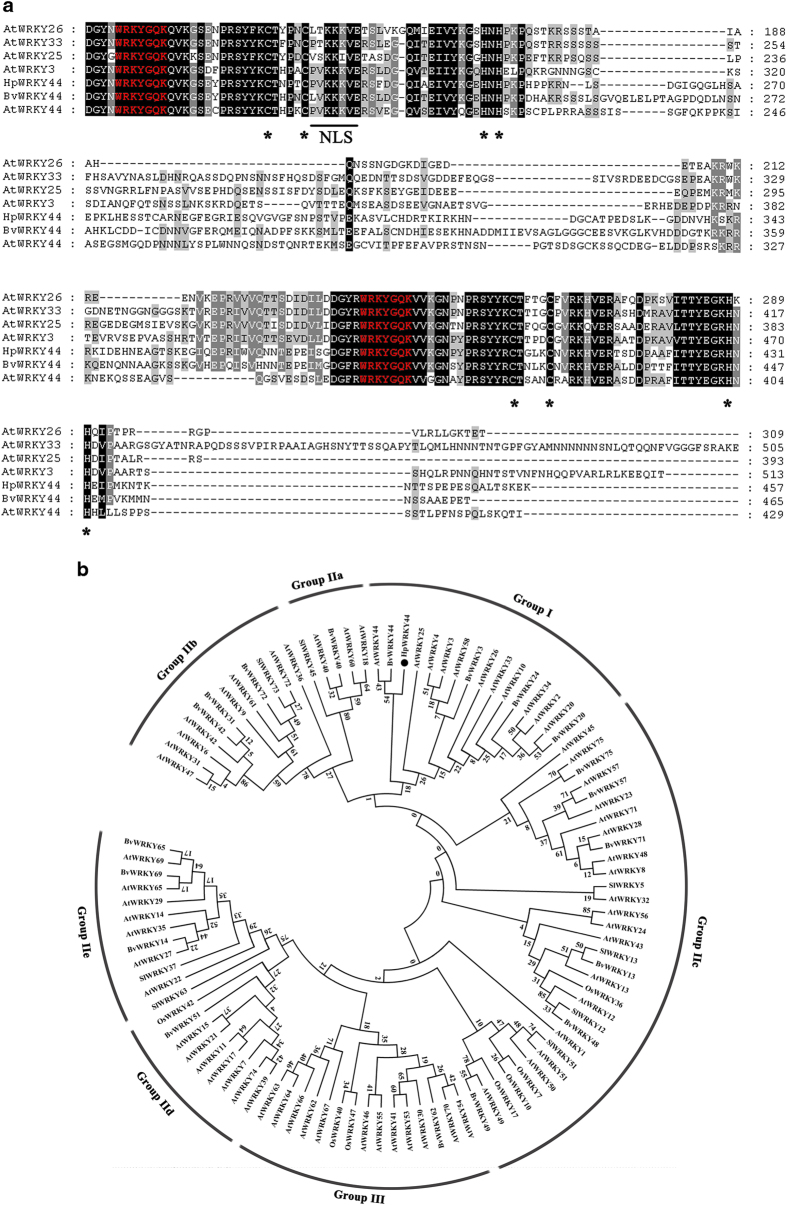
Bioinformatic analysis of HpWRKY44. (**a**) Multiple alignment of HpWRKY44 with sugar beet BvWRKY44, and *Arabidopsis thaliana* AtWRKY3, AtWRKY25, AtWRKY26, AtWRKY33 and AtWRKY44. Identical and similar amino acids are represented by black and gray shading, respectively. The two WRKY motifs and the zinc-finger structures are indicated by red letters and asterisks, respectively. A nuclear localization signal (NLS) is underlined. (**b**) Phylogenetic tree of HpWRKY44, sugar beet, *Arabidopsis thaliana*, rice and tomato WRKYs. WRKYs are divided into three major groups and seven sub-families. HpWRKY44 (black circles), along with sugar beet, BvWRKY44, *Arabidopsis thaliana* AtWRKY25, AtWRKY44 and tomato SlWRKY5 cluster in Group I. The phylogenetic tree was constructed with MEGA5.0 using a bootstrap test of phylogeny with UPGMA test and default parameters.

WRKY TFs in plants have been implicated in the regulation of phenylpropanoids, alkaloids and terpenes, through their ability to regulate metabolite biosynthetic genes.^25–28^ Betalains are secondary metabolites and the *HpCytP450-like1* promoter contains a motif suitable for the binding of WRKY TFs (Table 1). This finding prompted further analysis of the relationship between WRKY TFs and betalain biosynthesis genes in pitayas. Our previous RNA-seq study identified a full-length *WRKY* gene that was up-regulated during color development in pitaya fruit pulp. The full-length sequence of this *WRKY* gene exhibited high similarity to AtWRKY44 (40%), and so was named *HpWRKY44*. The Open Reading Frame of HpWRKY44 is 1371 bp in length, and encodes a polypeptide of 457 amino acids, with a calculated molecular weight of 50.89 kDa, and a pI of 9.02. HpWRKY44 has two highly conserved amino acid sequences of WRKYGQK, which is a WRKY domain and a defining characteristic of WRKY TFs.^21^ HpWRKY44 also contains two putative zinc-finger motifs (C-X4-CX23–24-H-X1-H) (Figure 2a).

WRKY proteins are clustered into three major groups (I–III), and group II can be further subdivided into five subgroups (IIa–e).^16,21^ A phylogenetic tree was constructed using the amino acid sequence of HpWRKY44, sugar beet, *Arabidopsis thaliana*, tomato, and rice WRKYs. HpWRKY44 was clustered with Group I WRKYs, along with the BvWRKY44 from sugar beet, and the AtWRKY44, AtWRKY25 and AtWRKY33 from *Arabidopsis thaliana* (Figure 2b). Notably, AtWRKY33 and AtWRKY44 have been reported to act as transcriptional regulators of phenylpropanoid and indole alkaloid biosynthesis,^37,38^ further indicating a potential role for HpWRKY44 in the biosynthesis of secondary metabolites in pitaya.

### Expression of *HpCytP450-like1* and *HpWRKY44* during color development in pitaya fruit pulp

**Figure 3:**
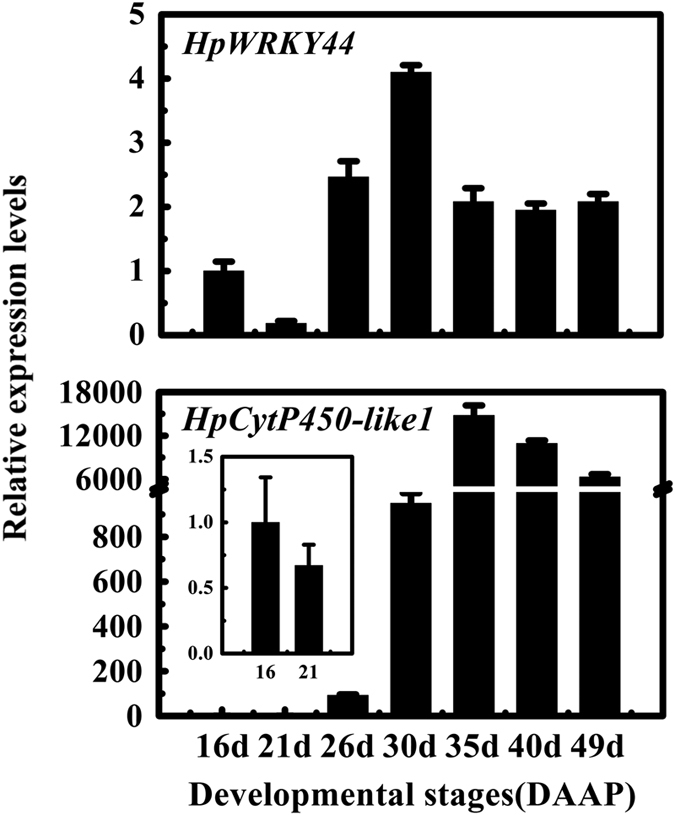
Expression pattern of *HpCytP450-like1* and *HpWRKY44* during fruit pulp coloration. Fruits were sampled at 16^th^, 21^st^, 26^th^, 30^th^, 35^th^, 40^th^ and 49^th^ days after artificial pollination (DAAP). The expression level of each gene is expressed as a ratio relative to 16 DAAP, which was set at 1. Each value represents the mean±s.e.m. of three replicates (*n*=3).

The expression level of *HpCytP450-like1* and *HpWRKY44* during the coloration of the pulp was determined by RT-qPCR in order to examine their relationship to betalain biosynthesis during fruit development. Results indicated that the transcript levels of *HpCytP450-like1* and *HpWRKY44* were relatively low from 16 to 21 DAAP (Figure 3). Consistent with the accumulation of betalains at 30 DAAP, however, both *HpCytP450-like1* and *HpWRKY44* transcript levels increased by approximately 910- and 4.1-fold of the initial level at 30 DAAP, respectively. This elevation in expression was coincident with the increase in the red color development of the pulp (Figure 3).

### Nuclear localization of HpWRKY44 and trans-activation ability

**Figure 4:**
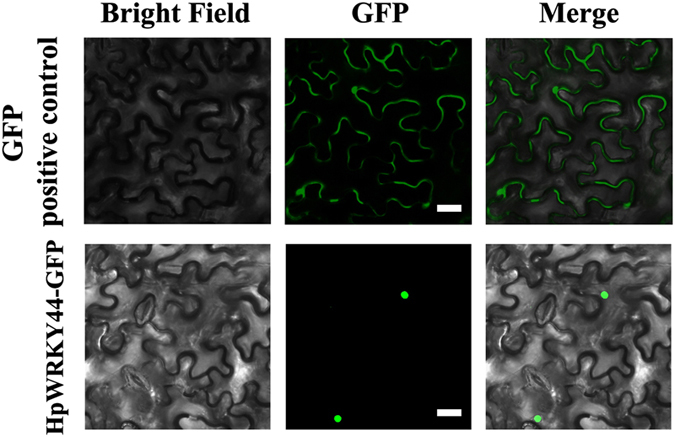
Subcellular localization of HpWRKY44 in tobacco leaves. *Agrobacterium tumefaciens* carrying HpWRKY44-GFP or a GFP positive control vector were infiltrated into tobacco leaves. After 48 h, the fluorescence of HpWRKY44-GFP protein was localized exclusively in the nucleus, while the fluorescence of the GFP positive control was distributed in both the nucleus and cytoplasm. Bar=30 μm.

WRKYs, like other TFs, are typically nuclear-localized proteins that possess transcriptional activity.^19,20,39,40^ Sub-cellular localization of HpWRKY44 was determined in tobacco leaves using a GFP-tagged HpWRKY44 protein driven by the CaMV 35S promoter. As shown in Figure 4, the HpWRKY44-GFP fusion protein was localized to the nucleus, while for the positive control, its GFP signal was observed around the cytoplasm and the nucleus.

**Figure 5:**
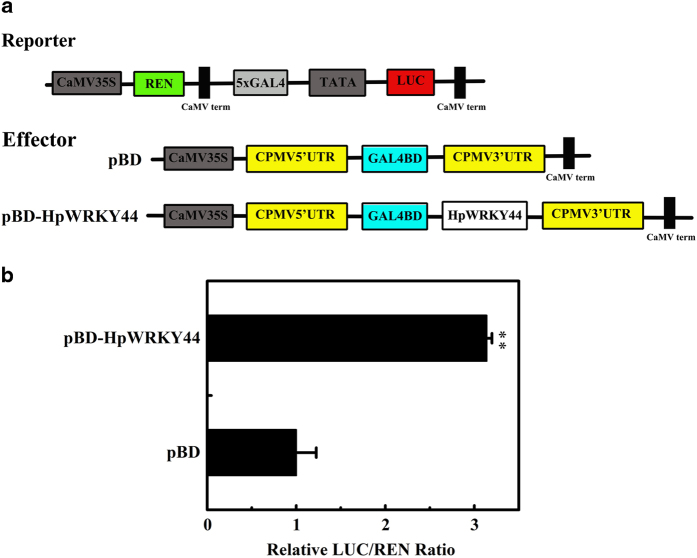
Analysis of transcriptional activity of HpWRKY44. (**a**) Reporter and effector constructs. The dual luciferase reporter construct contained the firefly luciferase (LUC) reporter gene fused with five copies of the GAL4 DNA-binding element (5×GAL4) plus the mini-35S (TATA box). The Renilla luciferase (REN) driven by CaMV 35S in the same vector was used as an internal control. The effector plasmid contained the HpWRKY44 gene fused to the yeast GAL4 DNA-binding domain (GAL4BD) driven by CaMV35S, CPMV, (Cowpea mosaic virus). (**b**) Transcriptional activation activity of HpWRKY44. The trans-activation ability of HpWRKY44 was assessed as the ratio of LUC to REN. Each presented value represents the mean±s.e.m. six biological replicates (*n*=6). The ratio of LUC/REN of the empty pBD vector was used as calibrator and set at 1. ** indicates a significant difference between the sample (transcriptional activator vector) and the control (empty pBD vector) at *P*<0.01, based on the Student’s *t*-test.

The transcriptional activity of HpWRKY44 in plant cells was also analyzed using a dual-luciferase reporter system (Figure 5a). Results of the analysis indicated that compared with the negative control pBD, HpWRKY44 obviously increased the value of the LUC/REN ratio (Figure 5b). These results indicate that HpWRKY44 is a nuclear protein and may potentially act as a transcriptional activator.

### Interaction of HpWRKY44 with the W-box in *HpCytP450-like1* promoter *in vitro*

**Figure 6:**
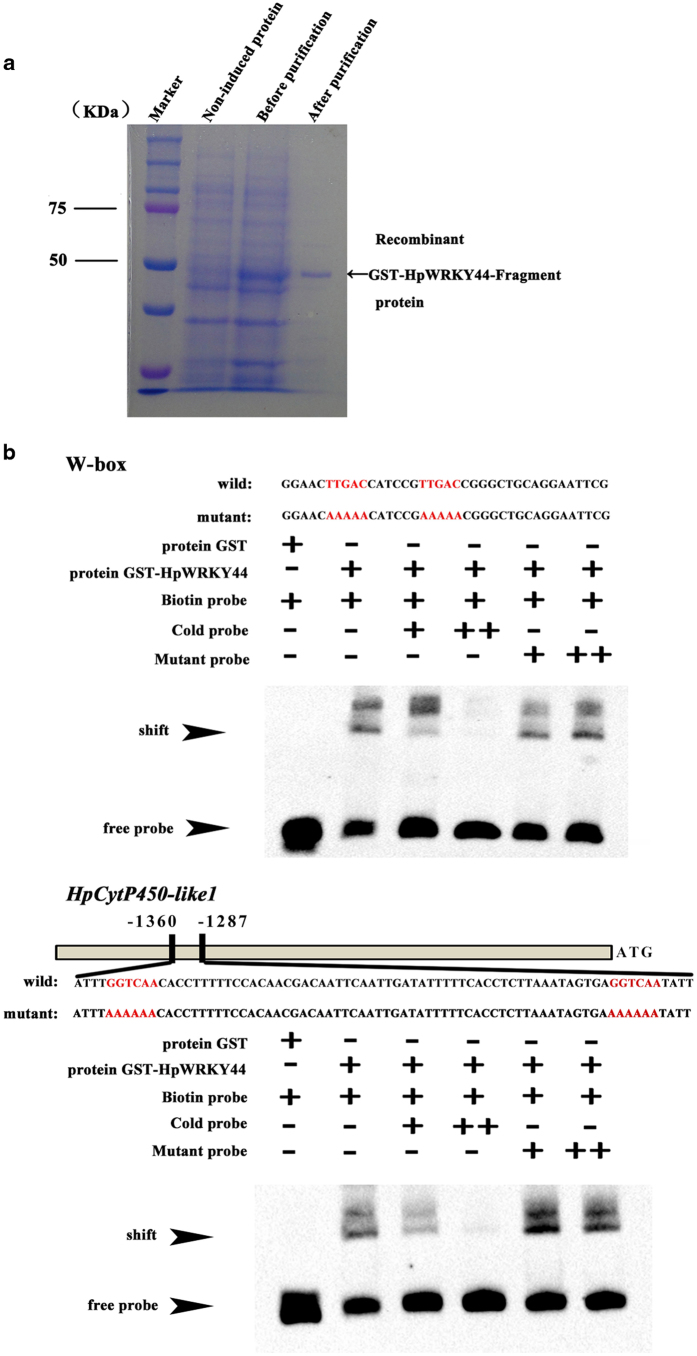
Electrophoretic mobility shift assay (EMSA) demonstrating the *in vitro* binding of HpWRKY44 to a W-box element and the *HpCytP450-like1* promoter containing a W-box element. (**a**) SDS–PAGE gel stained with Coomassie blue demonstrating affinity purification of the recombinant HpWRKY44 protein used for the EMSA. (**b**) EMSA. Biotin-labeled DNA probe from the promoter or mutant probe was incubated with GST-HpWRKY44 protein, and the DNA-protein complexes were separated on a 6% native polyacrylamide gel. GST protein alone was used as the negative control.+or ++ indicate increasing amounts of unlabeled or mutant probe used for competition and testing of binding specificity. Arrows indicate the position of the shifted bands.

Previous studies have reported the direct binding of WRKY TFs to the W-box motif within target gene promoters.^16,19,20,41^ For example, the WRKY TF, AaGSW1, of *Artemisia annua* can directly bind to W-box motifs in the *CYP71AV1* promoter.^42^ In the current study, the interaction between HpWRKY44 and the W-box of *HpCytP450-like1* promoter was characterized by EMSA. Firstly, glutathione S-transferase (GST)-HpWRKY44 fusion protein was prokaryotic induced and purified successfully (Figure 6a). Results indicated the formation of DNA-protein complexes with reduced migration when the recombinant GST-HpWRKY44 protein was mixed with the biotin-labeled DNA probe with two W-box motifs or with the *HpCytP450-like1* promoter containing a single W-box motif (Figure 6b). Formation of the DNA-protein complexes was effectively abolished when the corresponding unlabeled probes were added to the mixture as a cold competitor. but was not abolished when mutated W-box probes were used in the assay (Figure 6b). As expected, the mobility shift was also absent when the empty GST protein were incubated with biotin-labeled probes (Figure 6b). Collectively, these results indicate that HpWRKY44 is capable of binding to the W-box motif present in the promoter of *HpCytP450-like1*.

### HpWRKY44 activates the transcription of *HpCytP450-like1in vivo*

**Figure 7:**
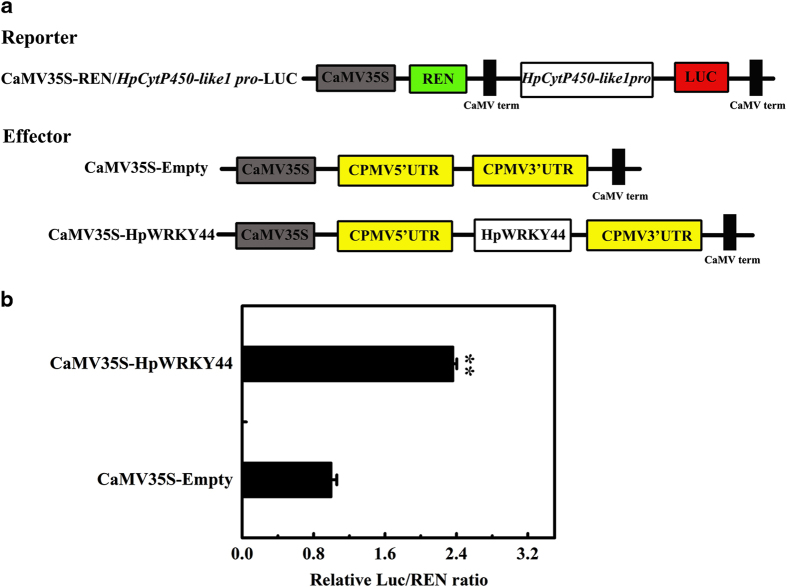
HpWRKY44 directly activates the expression of *HpCytP450-like1*. (**a**) Diagrammatic illustrations of the reporter and effector constructs used in the transient dual-luciferase reporter assay in tobacco leaves. LUC, firefly luciferase; REN, renilla luciferase; CPMV, Cowpea mosaic virus. (**b**) HpWRKY44 trans-activates the *HpCytP450-like1* promoter. The activation was assessed by the ratio of LUC to REN. The ratio of LUC/REN of the empty vector plus promoter was used as a calibrator and set at 1. Each presented value represents the mean±s.e.m. six biological replicates (*n*=6). ** Indicates statistically significant differences at *P*<0.01, as determined by Student’s *t*-test.

The ability of *HpCytP450-like1* to activate the transcription of HpWRKY44 was determined using transient dual-luciferase assays in tobacco leaves (Figure 7a). HpWRKY44 was inserted into the pEAQ vector to serve as an effector, and the empty pEAQ was included as a control (Figure 7a). As shown in Figure 7b, overexpression of HpWRKY44 significantly increased the LUC/REN ratio of the reporter containing *HpCytP450-like1* relative to the corresponding empty control. These results indicate that HpWRKY44 activated the transcription of *HpCytP450-like1* and supports the premise that HPWRKY44 has a functional role in the regulation of *HpCytP450-like1*. In red beets, a MYB1 TF, BvMYB1, has been reported to be involved in betalain biosynthesis, as silencing of BvMYB1 results in the down-regulation of betalain biosynthetic genes and pigmentation.^14^ BvMYB1 can also target the *CYP76AD1* promoter.^14^ Interestingly, MYB and AREB/ABF TFs binding sites are also present in the promoter of *HpCytP450-like1*. Therefore, whether or not *HpCytP450-like1* is also targeted by MYB and AREB/ABF TFs needs to be examined. The identification of additional TFs involved in the regulation of betalain biosynthesis genes in pitaya fruit also need to be identified. Moreover, it has been well documented that regulatory proteins, such as TFs, seldom act alone. Numerous studies have demonstrated that WRKY TFs physically interact with a wide range of proteins that have functional roles in signaling, transcription and chromatin remodeling. These protein interactions can affect the DNA-binding and transcription-regulatory activity that WRKY TFs have with their targets genes.^43^ For example, MaWRKY1 and MaWRKY2 in banana fruit cooperate with a NAC TF, MaNAC5, to activate the expressions of a specific set of *PR* genes associated with disease response.^44^ In addition, MaVQ5 in banana fruit antagonizes MaWRKY26 in the activation of JA biosynthesis in response to cold stress.^40^ Therefore, in future studies, it would be interesting to determine whether or not HpWRKY44 can coordinate with MYB AREB/ABF TFs, or other proteins to regulate betalain biosynthesis.

## Conclusions

In summary, a Group I WRKY TF, HpWRKY44, was cloned and characterized from pitaya fruit. HpWRKY44 activated *HpCytP450-like1* expression by binding to its promoter. To the best of our knowledge, this is the first report of the involvement of WRKY TFs in regulating *HpCytP450-like1* in fruits. Overall, our findings provides new insights into the transcriptional regulation of genes associated with betalain biosynthesis in pitayas; which are economically important and nutritious fruit.

## Figures and Tables

**Table 1:** Main regulatory motifs found within the *HpcytP450-like1* promoter

*Factor or site name*	*Site*	*Signal sequence*	*Function*
ABRE	719 (−);107(−)	TACGTG CGTACGTGCA	cis-acting element involved in the abscisic acid responsiveness
ACE	852(+)	AAAACGTTTT	cis-acting element involved in light responsiveness
ARE	225 (+)	TGGTTT	cis-acting regulatory element essential for the anaerobic induction
ATGCAAAT motif	688(−)	ATACAAAT	cis-acting regulatory element associated to the TGAGTCA motif
Box I	508 (+)	TTTCAAA	Light responsive element
CAAT-box	244(−);703(−);1256(−)	CCAAT GGCAAT	Common cis-acting element in promoter and enhancer regions
CAT-box	1131 (+);1454(+)	GCCACT	cis-acting regulatory element related to meristem expression
CATT-motif	45(+);70(+)	GCATTC	Part of a light responsive element
CCAAT-box	1086 (+)	CAACGG	MYBHv1 binding site
CGTCA-motif	960(+)	CGTCA	cis-acting regulatory element involved in the MeJA-responsiveness
ERE	508(+)	ATTTCAAA	Ethylene-responsive element
G-Box	719(+);1156 (+)	CACGTA CACGAC	cis-acting regulatory element involved in light responsiveness
GARE-motif	292(+)	TCTGTTG	Gibberellin-responsive element
GATA-motif	849(−)	AAGGATAAAG	Part of a light responsive element
Gap-box	1356(−)	CAAATGAA(A/G)A	Part of a light responsive element
HSE	1485(−)	AAAAAATATC	cis-acting element involved in heat stress responsiveness
LAMP-element	1418(+)	CTTTATCA	Part of a light responsive element
MBS	318(+)	TAACTG	MYB binding site involved in drought-inducibility
MRE	340(+);345(+)	AACCTAA	MYB binding site involved in light responsiveness
Skn-1_motif	1480(+);546(−);959(+); 896(−);983(−)	GTCAT	cis-acting regulatory element required for endosperm expression
TATA-box	952(−);156(−);332(−); 573(−);619(−);1034(+);747(−);635(+)	TATTTAAA TATA TATAAA TATAA	Core promoter element around −30 of transcription start
TGACG-motif	960(−)	TGACG	cis-acting regulatory element involved in the MeJA-responsiveness
W box	1174(−);1291(−); 1351(−)	TTGACC	WRKY binding site
Circadian	585(+);1147(+)	CAANNNNATC	cis-acting regulatory element involved in circadian control
