# Supplementary material for: The WRKY transcription factor HpWRKY44 regulates CytP450-like1 expression in red pitaya fruit (Hylocereus polyrhizus)
Source: Hortic Res. 2017 Aug 2;4:17039–. doi: 10.1038/hortres.2017.39 (PMC5539414; doi:10.1038/hortres.2017.39)
Supplement: Supplementary Information [file hortres201739-s1.doc]

**Supplementary Table S1.** Summary of primers used in this study

| **Assay** | **Primer sequence** | **Restriction**  **Site** |
| --- | --- | --- |
| **Full length**  **cloning** | ***HpWRKY44 For*** ATGTTGTATTTGTTCAGGATGGAGTTG  ***HpWRKY44 Rev*** CTATTTCTCTTTGGAAGTAAGTGCTTG |  |
| **Promoter**  **cloning** | ***HpCytP450-like-sp1*** GGCCCGGAGGTAAGGGGGGTCTTCTTCTTTTG  ***HpCytP450-like-sp2*** TGACCACATTAAACCCTAGCTTCACAATTTGA  ***HpCytP450-like-sp3*** TGAGGCAAAGATGAAAANNNNNNNNNTTGGGC |  |
| **Subcellular**  **localization** | ***HpWRKY44-GFPFor*** ttctgcccaaatcgcgATGTTGTATTTGTTCAGGATGGAGTTG  ***HpWRKY44-GFPRev***  tagtcataccggtcgcTTTCTCTTTGGAAGTAAGTGCTTG | ***NruI***  ***NruI*** |
| **RT-qPCR** | ***HpWRKY44 For*** CAATAAGGGTTAAGCCTGCAGTG  ***HpWRKY44 Rev*** TGGACATGGATTCGAGACCTGA  ***HpCytP450-like1 For*** CATACCCAACTCAGTCACGGC  ***HpCytP450-like1 Rev*** CTAAGGCTAGAGCAAGCATCGAG  ***Actin For*** CTTCCATACCAATGAATGAGG  ***Actin Rev*** AACCGCCAAGAGTAGTTCTG |  |
| **Fusing GST** | ***pGEX-4T-1-HpWRKY44-For*** g*gttccgcgtggatcc*ATGCCCTCTTATGATGGATATAATTGGA  ***pGEX-4T-1-HpWRKY44-Rev***  agtcacgatgcggccgc CTAATAGCTTCTGGGGTATGGGTTTCCC | ***BamHI***  ***NotI*** |
| **Dual LUC**  **assay** | ***pEAQ-HpWRKY44For*** caaattcgcg*accggt*ATGTTGTATTTGTTCAGGATGGAGTTG  ***pEAQ-HpWRKY44Rev***  agttaaaggc*ctcgag*TTTCTCTTTGGAAGTAAGTGCTTG  ***pBD-HpWRKY44For***  tcgccgaccggtaggcct ATGTTGTATTTGTTCAGGATGGAGTTG  ***pBD-HpWRKY44Rev***  aaccagagttaaaggcct TTTCTCTTTGGAAGTAAGTGCTTG  ***0800- HpCytP450-like1 pro For***  Tatagggcgaattgg AGTTGATTGACATTTATCTCTACCA  ***0800- HpCytP450-like1 pro Rev***  Ttggcgtcttccatgg GCTTTTGGGAGGGGGGGGGGGCTAG | ***AgeI***  ***XhoI***  ***Stu I***  ***Stu I***  ***KpnI***  ***NcoI*** |

**Supplementary Text S1.** Nucleotide sequence of *HpCytP450-like1* promoter. W-box (TTGACC) is indicated in box. Translation start site (ATG) is shown in red.

*> HpCytP450-like1*

AGTTGATTGACATTTATCTCTACCATGATAATTTTATCGATATTTTTTGTCATTAAAATCACTCAGTCGCCTCGCCACTAAAATAATTTTAACAGTTTGGATCGAAACTTTATCATCAAATAAAAAATATTAAAATAGGCTCAAATTCAGAGTTTGAGCTCGGTATTTTTTCATTT**GGTCAA**CACCTTTTTCCACAACGACAATTCAATTGATATTTTTCACCTCTTAAATAGTGA**GGTCAA**TATTTTTCATCACTAGAATAATTTCAACAAATTGGATTGAAACTTGATCATCGAATATGAAAGATTAAAATAAGCTCAAATTCATATTTTGAGTACAATATTCCTTCATTT**GGTCAA**TATTTTATCTGCCACGACAATTTAATCGATATTTCTCGCCACTTAAACAATTAAGTTGATTTTTTAGTCACTAAAACAATTTCAACGGTTTAGATCGAAACTCGGCTATCCAGACAGAAGTGTTCAAAACACAAATATAAATTTTAAGCTCAAAATTCCTTCGTTTGTTAAACATTTATCTCCACTATGACCATTCAGTCGATATTTTTCGTCATTTAAATAATTCAATTGACATTTTTTCTTTTTCATGAAAAATGATTTATTGTGCACCAAATGACCAAAAATGTGACATAAGACTCAATTTTACTTGGAAAAACGTTTTCCATAGAAAATGATTTCTTGGAAAAACTGTTTTCCGCAGAAAATGATTTTTCGAGAAAACAACTTCCTTTGGAACAAAACACTATCTTAATTAAAACATCGTATATGTGCTTTGGAAGGACACGCACCACGTATATGATTTTTTATTGGACATTTCATTTGTATATTTCAATAAACTTTTTTGTAGACAACATCAATCTCTTTATCCTTAATTATAAACCCTTAAAAATTATATTTCACATCTTTTCATTATGTCAACAAAAAAATCCTATGAGATATATAGCATTTTGCACTGAATAAAAATGACCATATTTAAATTAAAAAAAAATCATGTACAATTTCAAATTTCAGCATTTTTTTTTAATAATTGAAGCCAAAGGCGGGCCATGGCAGGAGCAGTAAAAAAGATAACTTATTTGGCCGGCACTCGCAAGTAATCAAAGGATCGCACCACCACCGCTGGTCTGTAAATAGTATTGTCCAGCCTAAAACTGATCCATCAACCTAACCTAAATTTTATAGAATAGAGTAACTGGCTCAGCTCAAGAGATCGATCTGTTGTCAAACCTCAGCCTCACTAGCTCATTCAAAATAATTTTCACTATTGCCAAATCCCATCTTATGGTTTTCTCCAAACATTATGCACACCCATGCCTAGCTGGATTCCCTTTCCTACCTACATGCACATGTGTGTATATAAATGTATGTCTGCAAATGTTCGCTTATCAAGTCAAGGTGAACGTACGCCAACAAGCCTGCATGTATTACATGCATACGCATTCCTAGCTAGCTAAAATTCCCTGCATTCTCTCTCTCCTTCTCTCATCCTAGCCCCCCCCCCCTCCCAAAAGCATG
